# Amelioration of intestinal ischemia reperfusion injury by diacerein via regulation of inflammasome/caspase-1/IL-1β and Wnt/β-catenin pathways in juvenile rats

**DOI:** 10.1007/s00210-025-04581-2

**Published:** 2025-09-15

**Authors:** Marwa Monier Mahmoud Refaie, Nada Amgad Mohamed Abdel Majeed, Sayed Shehata, Asmaa A. Muhammed, Salma M. Hassan, Hoda S. Sherkawy, Fatma F. Ali, Mohamed Rabie Saad, Mousa Mohsen, Shereen Mohammed Mohammed Elsaghir, Enas Fathy, Olivia N. Beshay

**Affiliations:** 1 Department of Medical Pharmacology, Faculty of Medicine, Minia University, 61511 Minia, Egypt; 2 Department of Histology and Cell Biology, Faculty of Medicine, Minia University, 61511 Minia, Egypt; 3 Faculty of Physical Therapy, Lotus University, 61786 Minia, Egypt; 4 Department of Cardiology, Faculty of Medicine, Minia University, 61511 Minia, Egypt; 5 Department of Medical Physiology, Faculty of Medicine, Aswan University, Aswan, 81528 Egypt; 6 Department of Human Anatomy and Embryology, Faculty of Medicine, Minia University, 61511 Minia, Egypt; 7 Department of Medical Biochemistry, Faculty of Medicine, Aswan University, Aswan, 81528 Egypt; 8 Department of Medical Physiology, Faculty of Medicine, Minia University, 61511 Minia, Egypt; 9 Department of Biochemistry, Molecular Biology and Physiology, Faculty of Medicine, Mutah University, Al-Karak, Jordan; 10 Department of Surgery, Faculty of Medicine, Aswan University, Aswan, 81528 Egypt; 11 Department of Pediatrics, Faculty of Medicine, Aswan University, Aswan, 81528 Egypt; 12 Department of Internal Medicine, Faculty of Medicine, Minia University, Minia, 61511 Egypt; 13 Department of Cardiology, Faculty of Medicine, Aswan University, 81528 Aswan, Egypt; 14 Department of Biochemistry, Faculty of Pharmacy, Minia University, 61511 Minia, Egypt; 15 Minia National University, 61768 New Minia, Egypt

**Keywords:** Diacerein, Intestinal ischemia reperfusion, Interleukin-1β, Inflammasome

## Abstract

Intestinal ischemia reperfusion (II/R) is an abdominal critical case especially in neonates and during childhood affecting not only the intestinal tissue but also it could damage other remote organs including cardiac tissue even following surgical intervention. Immune homeostasis during II/R has a major role in controlling its progression. Thus, finding additive medical treatment besides the surgical one becomes an urgent need to keep the tissue. So that, we aimed to evaluate the possible ameliorative effect of diacerein (DIA) on II/R-induced injury in juvenile rats. Forty juvenile rats of Wistar albino species were randomly allocated into four different groups: sham group, DIA given group, II/R group via clamping superior mesenteric artery, DIA-treated group (50 mg/kg) with induction of II/R. Data of current model revealed a significant elevation of the measured cardiac enzymes, cleaved caspase-3, and nuclear factor kappa β (NF-κB) in the untreated ischemic group with disturbed oxidative stress parameters, accompanied with dysregulation of inflammasome/caspase-1/IL-1β and Wnt/β-catenin signaling cascades. Fortunately, upon co-administration of DIA, there is a significant decrease of cardiac enzymes, cleaved caspase-3, and NF-κB with normalization of oxidative stress parameters and regulation of inflammasome/caspase-1/IL-1β and Wnt/β-catenin pathways confirmed by marked mitigation of the histopathological changes. This effect of DIA is greatly attributed to its pharmacological properties including IL-1β antagonist effect, anti-oxidant, anti-apoptotic, and anti-inflammatory properties. Thus, DIA could be considered as an adjuvant future medical therapy for those cases of II/R.

**Graphical abstract:**

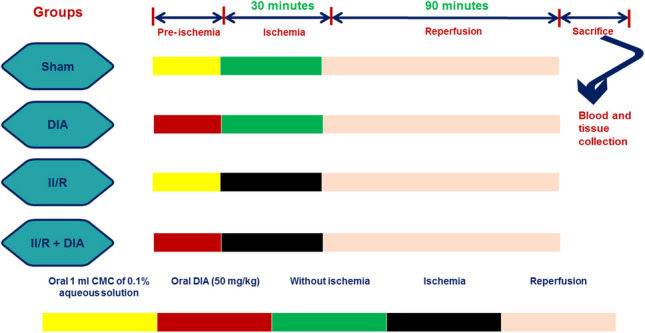

## Introduction

Immune dysregulation plays a crucial role in mediating the pathogenesis of ischemic disorders. Interleukin-1 beta (IL-1β) is one of the essential pro-inflammatory cytokines involved in intestinal and cardiac ischemia. It has a potent modulating action on the intestinal epithelial tight junction and increases the intestinal permeability. In addition, IL-1β level markedly increases after intestinal ischemia reperfusion (II/R) and activates macrophages in the early stages of intestinal ischemic injury (Al-Sadi et al. 2012; Monaco et al. 2021; Xue et al. 2022; Ruera et al. 2022). This is especially peculiar in neonates as the inflammatory process is more pronounced, compensatory mechanisms are still immature and the immune system is constantly developing. II/R in neonates and early childhood is coupled during various clinical conditions including acute mesenteric ischemia, neonatal necrotizing enterocolitis, volvulus, and incarcerated hernia (Grenz et al. 2012; Li et al. 2022; Archontakis‐Barakakis et al. 2025). Nevertheless, the blood supply restoration is accompanied by more formation of reactive oxygen species (ROS) that attack all intracellular components with marked release of the inflammatory mediators as tumor necrosis factor α (TNF-α), interleukin-1β (IL-1β), and nuclear factor kappa beta (NF-κB) (Tan et al. 2023; Cai et al. 2025).

During reperfusion, there is a harmful disruption of the mucosal barrier integrity allowing bacterial translocation from the intestine into the extra-intestinal sites (Deng et al. 2022; Li et al. 2022; Gadde et al. 2025). This results in endotoxemia, and development of systemic inflammatory response syndrome, and even occurrence of multiple organ dysfunction syndrome, especially those having a high proportion of the endothelial cells, such as the heart, lung, liver, and kidney (Ricardo-da-Silva et al. 2021; Akinrinde and Akinrinmade 2023; Chen et al. 2024). Intestinal ischemic injury is not localized to the intestinal issue, but it may have other harmful cardiovascular consequences. This is due to the associated serious oxidative stress processes, releasing of free radicals, inflammation, and endothelial dysfunction (Bala et al. 2022). The gut releases different pro-inflammatory cytokines including TNFα, IL-6, and IL-1β into the circulation, leading to intestinal and cardiac injury (Doudakmanis et al. 2021; Ji et al. 2023; Gültekin et al. 2024). Thus, it is assumed that proper inhibition of IL-1β receptors, inflammation, oxidative stress, apoptosis, and controlling inflammasome/caspase-1/IL-1β signaling cascade will mitigate II/R-induced damage. Furthermore, the Wnt/β-catenin pathway has been proved to be deeply involved in a variety of physiological and pathological processes as it is an essential player in embryonic development and tissue homeostasis controlling the expression of various mediators, plasminogen activator inhibitor-1, and renin-angiotensin system (Li et al. 2021). Besides that, there is a crosstalk between Wnt/β-catenin and NF-κB signaling cascade via a bidirectional manner, in that both pathways regulate each other reciprocally. Evidences suggest that Wnt/β-catenin pathway can downregulate the production of pro-inflammatory cytokines including inflammasome/caspase-1/IL-1β and TNF-α under various stimuli, via Wnt/β-catenin-mediated suppression of NF-κB activity that is responsible for the pathogenesis of ischemic injury (He et al. 2022; Zhang et al. 2024).

Diacerein (DIA) is a selective inhibitor of IL-1β used mainly in treating osteoarthritis and it could improve renal, cardiac, brain, testicular, and hepatic microcirculation during ischemia reperfusion-induced damage, anticancer toxicity, and diabetic cardiomyopathy (Torina et al. 2015; Abdel-Gaber et al. 2018; Chen et al. 2021; El-aziz Fathy et al. 2024; Refaie et al. 2024b; Ali et al. 2025). Based on the previously described data, there is a great association between inflammasome/caspase-1/IL-1β pathway, occurrence of oxidative stress, inflammation, apoptosis, and endothelial dysfunction during II/R injury. Meanwhile, DIA is suspected to have an ameliorative role in such model depending on its properties in blocking IL-1β, anti-inflammatory, anti-oxidant, and anti-apoptotic effects. This guided our attention to study the novel possible mitigating role of DIA in II/R-induced intestinal and cardiac injuries in juvenile rats with deep studying of the different responsible mechanisms including inflammasome/caspase-1/IL-1β and Wnt/β-catenin cascades.

## Study methodology

### Animals

Juvenile male Wistar albino rats weighing about 80–100 g were included in the current model delivered from the Egyptian National Research Center, Giza, Egypt. All juvenile rats were adequately fed on a standard diet of the experimental animals with chow and tap water then left to be acclimatized for a sufficient period about 2 weeks in clean stainless steel cages; three rats in each one with exposure to 12-h dark:light cycle and kept at 24 ± 2 °C. The Committee for Care and Use of Laboratory Animals of Faculty of Medicine, Aswan University, approved this study; No: Asw.Uni./1087/4/25 in accordance with EU Directive 2010/63/EU guidelines and ARRIVE guidelines.

### The used chemicals

DIA was from EVA Pharma, Egypt, and total antioxidant capacity (TAC) kits (Catalog # TA 2513) were from Biodiagnostic Co., Egypt. ELISA kits of the measured cardiac enzymes, troponin I, creatine kinase-MB (CK-MB), lactate dehydrogenase (LDH), inflammasome, NF-κB with catalog numbers (MBS2033695), (MBS722833), (MBS2515061), (MBS2033695), and (MBS453975) respectively, were from My BioSource Co., San Diego, CA, USA, and primary antibodies, including Wnt1 (Santa Cruz Biotechnology, USA, Catalog # sc-514531), IL-1β (Santa Cruz Biotechnology, USA, Catalog# sc-515598), and β-actin (Abcam, Catalog#ab8226), rabbit polyclonal anti-caspase-1, inflammatory marker (Catalog # A18646 from Abclonal, USA), rabbit polyclonal anti-caspase-3, apoptotic marker (Catalog # A11953 from Abclonal, USA), and rabbit monoclonal anti-β-catenin, adherens junction marker (Catalog # A19675 from Abclonal, USA). Ready Prep™ protein extraction kit and enhanced chemiluminescence substrate (Clarity™ Western ECL substrate) were supplied from Bio-Rad, USA, with catalog numbers (Catalog #163–2086) and (Catalog #170–5060), respectively.

### Study protocol

#### Animal groups

The rats were immediately allocated into four different groups (*n* = 10).

Sample size of 10 rats in each group was determined according to the previous studies, the pilot, and the expected mortality to obtain sufficient number of rats to provide a level of 95% power, effect size 0.7, and 0.05 significance using G Power 3.1 9.2 software (total samples = 40) (Noordzij et al. 2011; Charan and Biswas 2013).

- **Group 1:** Sham group: Rats were exposed to all surgical steps except vessel occlusion plus receiving 1 ml of carboxymethyl cellulose (CMC) of 0.1% aqueous solution.
- **Group 2:** DIA given group: Animals were exposed to all surgical steps except vessel occlusion plus receiving DIA (50 mg/kg) (Chen et al. 2021; Refaie et al. 2024b) single oral dose 3 h before starting the experiment.
- **Group 3:** II/R untreated group: Animals of this group received CMC 1 ml of 0.1% aqueous solution by an oral gavage single dose followed by abdominal sterilization using Betadine then laparotomy and occluding the superior mesenteric artery by bulldog clamps for 30 min as an ischemic period then reperfusion for 90 min.
- **Group 4:** II/R + DIA-treated group: Rats received DIA (50 mg/kg) by an oral gavage 3 h before starting the experiment followed byabdominal sterilization using Betadine then laparotomy and occluding the superior mesenteric artery by bulldog clamps for 30 min as an ischemic period then reperfusion for 90 min (Refaie et al. 2024a).

Dose of DIA was detected according to our previous pilot studies and other models as 50 mg/kg was the most effective dose. DIA was properly suspended in 0.1% aqueous solution of CMC before starting the experiment.

### Steps of surgery

Urethane hydrochloride (1 g/kg) was given as an anesthetic agent by intra-peritoneal route in single dose, followed by sterilizing the abdominal region using Betadine. An abdominal midline incision is performed followed by exposure and occlusion of the superior mesenteric artery by small bulldog clamps for 30 min then removal of these clamps for 90 min as a reperfusion period. Heating pad was used to preserve the rat body temperature at 37 °C. The sham group was subjected to the same surgical steps without vessel occlusion (Chen et al. 2021).

### Sample collection

Following 2 h of ischemia reperfusion period, rats were gently sacrificed by a cervical dislocation method under the effect of the given anesthetic agent, and blood samples were obtained from the exposed jugular neck veins and properly centrifuged to get clear sera. We carefully excised the jejunal part of the small intestine and the left ventricle. Sections were immediately fixed in 10% formalin for further histopathological studying and other parts of the tissue samples were deeply frozen at − 80 °C until used for the subsequent biochemical measurements, and western blotting evaluation. Tissue was adequately homogenized in 5 ml phosphate buffer solution then centrifuged for 20 min at 4000 rpm. The obtained samples of the supernatant were separated in Eppendorf tubes.

### Cardiac enzymes, inflammasome, and NF-κB ELISA measurement

The cardiac enzymes (CK-MB, LDH, and troponin I), inflammasome, and NF-κB were detected by the described ELISA method according to the attached manufacturers’ instructions. In brief, the microtiter plate of each measured protein was previously pre-coated with its specific antibody, followed by the addition of samples or standards that bound to its specific antibody in the already pre-coated wells. This reaction was terminated with the addition of sulfuric acid.

### The evaluated oxidative stress parameters

Membrane lipid peroxidation was measured depending on the level of thiobarbituric acid reacting substance which was equivalent to malondialdehyde (MDA), using a standard curve of 1,1,3,3-tetramethoxypropane (Buege and Aust 1978). Reduced glutathione (GSH) was evaluated calorimetrically based on the binding of sulfhydryl group with Ellman’s reagent resulting information of a yellow color that is measured at 405 nm (Moron et al. 1979). Also, TAC was detected colorimetrically depending on the reaction of the antioxidants in each sample with a known amount of hydrogen peroxide. The residual amount was evaluated colorimetrically at 510 nm.

### Histological examination of the intestinal and cardiac tissue

Following fixation, samples from jejunum and heart tissue sections of the left ventricle were immediately dehydrated using increasing concentrations of alcohol. Finally, the samples were cleaned using two rounds of xylol. The impregnation process involved immersing the sample in a pure soft paraffin at a temperature of 55 °C for 2 h. Subsequently, the samples were embedded in hard paraffin. Ultimately, thin sections of 5 µm in thickness had been created using a microtome. These sections were stained using hematoxylin and eosin (H&E) stain in order to examine the histological characteristics of the jejunum and cardiac muscles (Bancroft and Layton 2019).

For immunohistochemical study, rabbit polyclonal anti-caspase-1, inflammatory marker, rabbit polyclonal anti-caspase-3, apoptotic marker, and rabbit monoclonal anti-β-catenin, adherens junction marker, were used according to the attached manufactures’ instructions. In brief, jejunal and cardiac muscle sections were de-paraffinized in xylene and rehydration was done in descendant concentrations of alcohol; the sections were immediately dipped in 0.1% hydrogen peroxide for 15 min to avoid the endogenous peroxidase activity. After that, sections were adequately rinsed in a phosphate buffer saline then incubated in the ultra-vision block for about 5 min at the room temperature to avoid the non-specific background staining. The sections were incubated with the specific primary antibody: anti caspase-1 (1:100), activated anti-caspase-3 (1:200), and anti β-catenin (1:100) overnight at 4 °C. Then the sections were washed in the buffer three times for 5 min each one. After that, they were incubated for 30 min with the secondary antibody then washed again. Half an hour later, sections were incubated with Vecta Stain ABC kits (Avidin-Biotinylated horseradish peroxidase complex) and washed for about 10 min. Reaction was recognized by applying UltraVision ONE Detection System, HRP (Horseradish Peroxidase) Polymer, and DAB (diaminobenzidine) Plus Chromogen (Thermo Fisher Scientific, USA). After the reaction was completed, hematoxylin was used for counter staining then sections were properly dehydrated through ascendant grades of alcohol and clearance was done by using xylene. Cover slip by a mounting media was finally applied (Suvarna et al. 2018). Negative controls were adequately performed (figures not included) to exclude nonspecific binding with the secondary antibody (the same immunohistochemical staining protocol without incubation with the specific primary antibodies).

Villous height in H&E-stained jejunal sections were detected × 200. Also, the degree of distorted architecture of the cardiac muscles, loss of muscle striation, inflammatory cellular infiltrate, presence of pyknotic nuclei, and vascular congestion. The severity of the lesion was graded as follows: score 0 was for normal, score 1 for mild changes, score 2 for moderate changes, and score 3 for severe changes (Constantin and Tăbăran 2022). Moreover, the mean area fraction of anti-caspase-1, anti-cleaved caspase-3, and anti-β-catenin immunoreactivity × 400 was assessed using the software image J program (ij152-win-java8). Images were acquired using an Olympus digital camera (LC20, Olympus Co., Tokyo, Japan) coupled to an Olympus light microscope (BX51, Olympus Co.). Ten non-overlapping fields from each rat per group were randomly selected and assessed for the histological scoring and image analysis (Schneider et al. 2012). The histologist was totally unaware of the studied group assignments.

### Western blotting analysis of IL-1β and Wnt1

In compliance with the supplier’s guidelines, total protein in homogenized tissue samples of the intestine and heart was extracted employing the Ready Prep™ protein extraction kit. Total protein concentration was quantified in each sample via the Bradford assay (Kielkopf et al. 2020). Twenty micrograms of total protein per sample was mixed with an equal volume of 2 × Laemmli buffer and loaded onto the wells of sodium dodecyl sulfate–polyacrylamide gel electrophoresis. Proteins were separated in accordance with their molecular weights, blotted to polyvinylidene difluoride membranes, which were subsequently immersed in a blocking solution at room temperature for 1 h prior to being incubated for the entire night at 4 °C with primary antibodies against IL-1β, Wnt1, and β-actin. Following several washings, incubation membranes in the horseradish peroxidase-conjugated secondary antibody solution was carried out for 1 h at the room temperature. At last, the blots were developed with enhanced chemiluminescence substrate, and the signals were captured applying a CCD camera-based imager. Band intensities corresponding to the target proteins were quantified applying TotalLab Analysis software (Version 1.0.1, www.totallab.com); with normalization to the β-actin expression levels and results were expressed as a fold change with respect to the sham group.

### Statistical analysis of the data

The collected data was expressed as means ± SEM that was accurately analyzed by the one-way analysis of variance followed by Tukey’s test. *P* values < 0.05 were considered significant. GraphPad Prism software was used (version 5.01 for Windows, San Diego, CA, USA).

## Results

### Effect of DIA on the measured cardiac enzymes

Untreated ischemic group had a significant increase in the levels of cardiac enzymes including CK-MB, LDH, and troponin if compared to the sham group while DIA co-given group showed significant diminish of these cardiac enzymes if compared to the untreated ischemic group, *P* < 0.001 (Table 1).

**Table 1:** Effect of DIA on cardiac enzymes in II/R model

Groups	Troponin I (ng/ml)	CK-MB (U/l)	LDH (U/l)
Sham	14.6 ± 0.8	17.0 ± 0.6	178.0 ± 4.2
DIA	17.6 ± 0.5	20.8 ± 1.1	203.6 ± 10.5
II/R	49.3 ± 2.5^ab^	50.50 ± 1.6^ab^	426.0 ± 9.1^ab^
II/R + DIA	22.20 ± 1.0^ac^	34.50 ± 1.8^abc^	290.4 ± 2.7^ac^

Values are representation of 10 observations (*n*=10) in each group as means ± S.E.M. Results are considered significantly different when *P* < 0.05. ^a^Significant difference compared to sham group. ^b^Significant difference compared to DIA group. ^c^Significant difference compared to II/R group. DIA isdiacerein, II/R is intestinal ischemia reperfusion induced group

### Evaluation of oxidative stress parameters (GSH, TAC, and MDA)

The untreated II/R group elevated MDA tissue levels but diminished the serum TAC and tissue GSH if compared to sham group. DIA co-administration had the ability to reverse this effect if compared to II/R untreated group, *P* < 0.0001 (Table 2).

**Table 2:** Effect of DIA on oxidative stress parameters

Groups	Intestine GSH (µmol/g tissue)	**I**ntestine MDA (nmol/g tissue)	Cardiac GSH (µmol/g tissue)	Cardiac MDA (nmol/g tissue)	TAC (mmol/l)
Sham	419.0 ± 11.4	38.4 ± 1.4	497.7 ± 11.5	33.5 ± 1.9	0.9 ± 0.02
DIA	439.0 ± 11.0	37.1 ± 1.4	492.0 ± 19.9	34.0 ± 1.5	0.8 ± 0.02
II/R	213.0 ± 11.4^ab^	83.7 ± 2.3^ab^	213.0 ± 11.4^ab^	74.2 ± 2.4^ab^	0.4 ± 0.03^ab^
II/R + DIA	302.0 ± 20.6^abc^	54.8 ± 2.8^abc^	332.0 + 21.6^abc^	47.4 ± 2.1^abc^	0.7 ± 0.04^abc^

Values are representation of 10 observations (*n*=10) in each group as means ± S.E.M. Results are considered significantly different when *P*< 0.05. ^a^Significant difference compared to sham group. ^b^Significant difference compared to DIA group. ^c^Significant difference compared to II/R group. DIA is diacerein, II/R is intestinal ischemia reperfusion induced group

### Measurement of tissue levels of NF-κB and inflammasome

Untreated ischemic group significantly elevated the tissue levels of NF-κB and inflammasome in intestinal and cardiac tissue if compared to sham group. DIA (50 mg/kg) given group could significantly reduce their tissue levels if compared to II/R untreated group, *P* < 0.001 (Table 3).

**Table 3:** Effect of DIA on the tissue level of NF-κB and inflammasome

Groups	Intestinal inflammasome (ng/ml)	Intestinal NF-κB (ng/ml)	Cardiac inflammasome (ng/ml)	Cardiac NF-κB (ng/ml)
Sham	29.3 ± 1.2	26.4 ± 1.2	25.3 ± 1.4	26.8 ± 1.3
DIA	30.2 ± 1.1	27.6 ± 1.5	29.7 ± 1.7	29.7 ± 1.2
II/R	84.4 ± 1.4^ab^	79.9 ± 2.2^ab^	85.2 ± 3.1^ab^	70.4 ± 1.9^ab^
II/R + DIA	60.0 ± 2.7^abc^	47.3 ± 2.0^abc^	66.5 ± 1.7^abc^	43.1 ± 1.5^abc^

Values are representation of 10 observations (*n* = 10) in each group as means ± S.E.M. Results are considered significantly different when *P* < 0.05. ^a^Significant difference compared to sham group. ^b^Significant difference compared to DIA group. ^c^Significant difference compared to II/R group. DIA is diacerein, II/R is intestinal ischemia reperfusion induced group

### Light microscopic results

#### Effect of DIA treatment on histological changes in jejunal sections

**Fig. 1:**
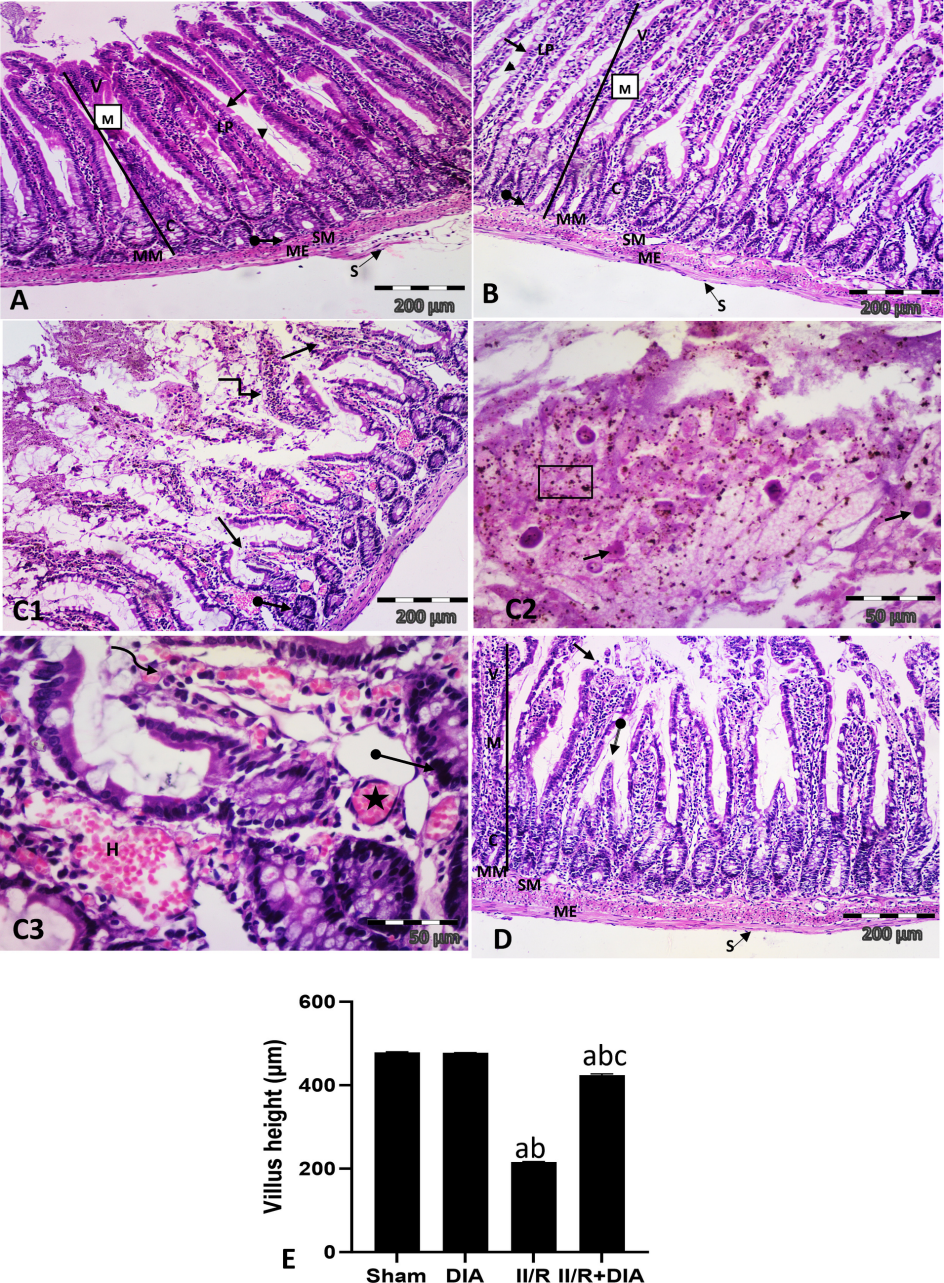
Representative photomicrographs of a cross sections in the jejunum of juvenile male Wister albino rat of (**A **& **B**) sham and DIA groups respectively showed normal histological structure of the jejunal wall formed of mucosa (M), submucosa (SM), muscularis externa (ME), and serosa (S). The mucosa of jejunum consists of villi (V) that appear as finger-like projections, tubular crypts of Liberkuhn (C), and thin muscularis mucosa (MM). The villi formed of core of connective tissue lamina propria (LP), covered by simple columnar epithelium with apical brush border (arrows), and goblet cells (arrow heads). Notice, pyramidal Paneth cells at the base of the crypt (oval arrows). (**C1**–**3**) II /R group: (**C1**) showing patchy erosions of the apical parts of most villi (arrows), other sloughed in lumen (elbow arrows), and distortion of crypts (oval arrow). (**C2**, **3**) A higher magnification showed detached acidophilic cells (arrows), and hemosiderin granules (square) in gut lumen, pyknotic nuclei line the crypt (oval arrow), dilated congested blood vessels (asterisks), extravasation of RBCs with inflammatory cellular infiltration (curved arrow), and intra-villous hemorrhage (H). (**D**) II /R + DIA group showed the normal structure of jejunal wall; mucosa (M), submucosa (SM), muscularis externa (ME), and serosa (S), almost normal villi (V), crypts (C), muscularis mucosa (MM) except for focal erosions of apical part of some villi (arrow), and few short villi (oval arrow). (H&E × 100, C2,3 × 400). (**E**) Semiquantitative analysis of villus height. Values are representation of 10 observations (*n* = 10) in each group as means ± S.E.M. Results are considered significantly different when *P* < 0.05. ^a^Significant difference compared to sham group. ^b^Significant difference compared to DIA group. ^c^Significant difference compared to II/R group. DIA is diacerein; II/R is intestinal ischemia reperfusion-induced group

The H&E-stained jejunal sections of sham control and DIA groups showed the normal histological structure of the jejunal wall formed of four basic layers: mucosa, submucosa, muscularis externa, and serosa. The mucosa consisted of finger-like projections called villi, tubular crypts of Liberkhun, and muscularis mucosa formed of thin layer of smooth muscle. The villi formed of a connective tissue core, lamina propria, covered by single layer of absorptive columnar cells with oval basal nuclei and apical acidophilic brush border, and goblet cells. Pyramidal Paneth cells were noticed at the base of the crypt (Fig. 1A, B). On the contrary, the II /R group revealed various histological changes in the form of patchy erosion of the apical parts of most villi, while other villi were sloughed in the lumen. Additionally, detached acidophilic cells, and hemosiderin granules were noticed in gut lumen. The crypts were distorted and the lining cells appeared darkely stained with pyknotic nuclei. Furthermore, dilated congested blood vessels, extravasation of RBCs with inflammatory cellular infiltration, and intra-villous hemorrhage were noticed (Fig. 1C1, 2, 3). On the other hand, the II /R+DIA-treated group exhibited improvement in the structure of the jejunum except for focal erosions of apical part of some villi, and few short villi (Fig. 1D).

Morphometric results showed that the II /R group had a significant decrease in the mean villus height compared to sham and DIA groups (both *P*<0.0001), while co-treatment with DIA showed significant increase compared to II /R group (*P*<0.0001) (Fig. 1E).

#### Effect of DIA treatment on histological changes in cardiac muscle sections

**Fig. 2:**
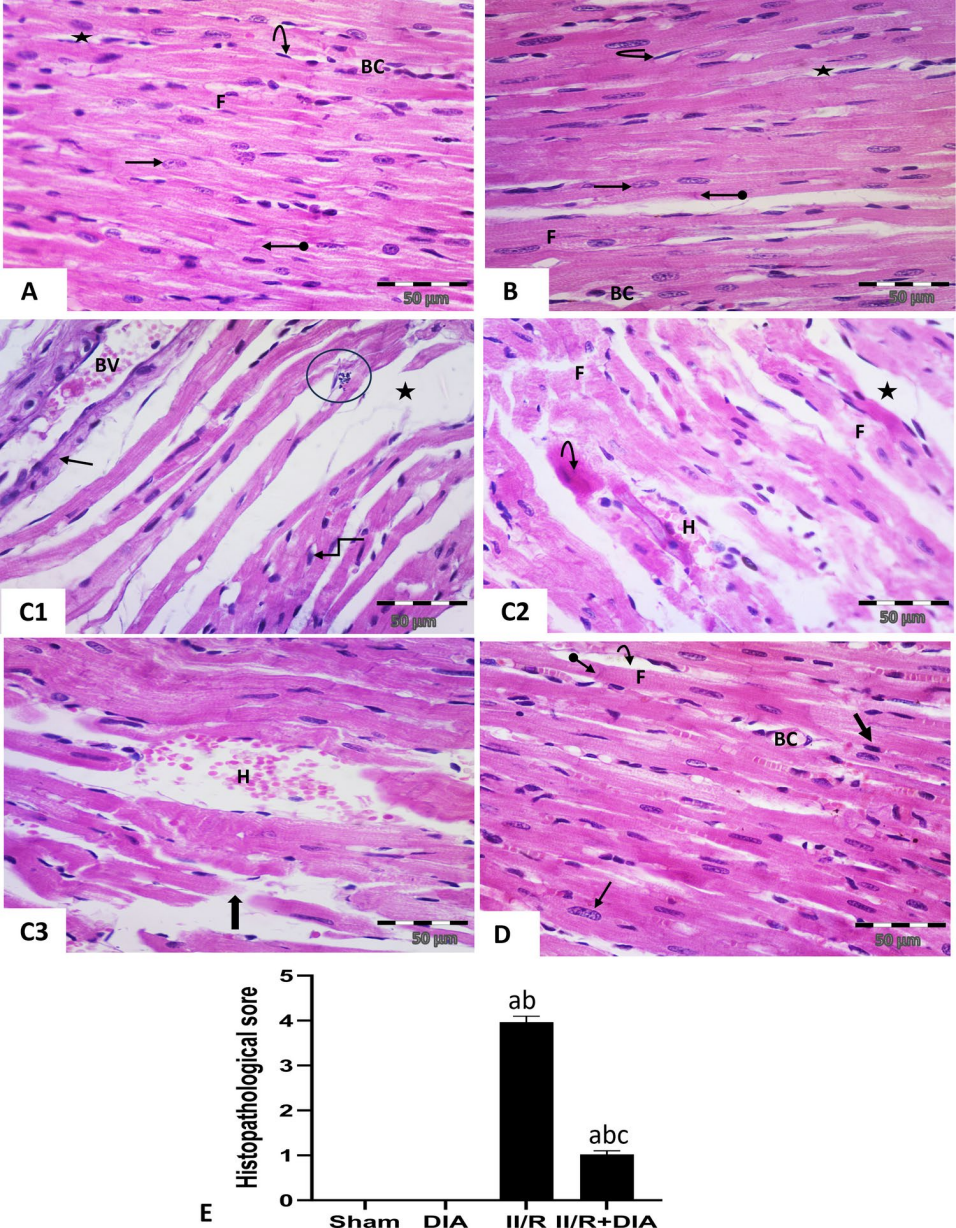
Representative photomicrographs of longitudinal sections of juvenile male Wister albino rat cardiac muscle of (**A** & **B**) sham and DIA groups respectively showing normal histological structure formed of branched striated acidophilic muscle fibers (F) with single central oval vesicular nuclei (arrows), intercalated discs (oval arrows), surrounded with thin endomysium (asterisk), and separated with dense flattened nuclei of fibroblasts (curved arrows), and few blood capillaries (BC). (**C1–3**) II / R group: (**C1**) showing wide space between myofibrils (asterisk), thin fibers with decreased acidophilia (arrow), darkly stained nuclei surrounded with white halo (elbow arrow), dilated blood vessels (BV), and inflammatory cellular infiltration (circle). (**C2**, **3**) showing fragmented fibers (F), homogenous hyperacidophilic fibers with peripheral darkly stained nucleus (curved arrow), discontinuous fibers (thick arrow), and interfibrillar hemorrhage (H). (**D**) II /R + DIA group showing approximately normal histological appearance of striated muscle fiber (F) with central oval vesicular nuclei (thin arrow), intercalated disc (oval arrow), minimal interstitial space (curved arrow), and decrease congestion of blood capillaries (BC). Some pyknotic nuclei (thick arrow) are still seen. (H&E × 400). (**E**) Histopathological scores. Values are representation of 10 observations (*n* = 10) in each group as means ± S.E.M. Results are considered significantly different when *P* < 0.05. ^a^Significant difference compared to sham group. ^b^Significant difference compared to DIA group. ^c^Significant difference compared to II/R group. DIA is diacerein; II/R is intestinal ischemia reperfusion induced group

Histological study of longitudinal sections of cardiac muscle of the left ventricle in sham and DIA groups revealed normal histological structure formed of branched and anastomosed striated acidophilic muscle fibers with single central oval vesicular nuclei, intercalated discs appeared between the adjacent fibers. Fibers were surrounded by thin endomysium containing fibroblasts with dense flattened nuclei, and few blood capillaries (Fig. 2A, B). While the II /R group showed wide interstitial space between myofibrils, thin elongated, fragmented, discontinuous fibers with decreased acidophilia, darkly stained nuclei surrounded with white halo. Furthermore, unlike sham and DIA groups, this group showed markedly dilated blood vessels and inflammatory cellular infiltration. Other fibers appeared homogenous with hyper-acidophilic sarcoplasm. Moreover, interfibrillar hemorrhage was noticed (Fig. 2C1, 2, 3). On the contrary, the II /R + DIA-treated group showed approximately normal histological appearance of striated muscle fiber with central oval vesicular nuclei, intercalated disc, minimal interstitial space, and decrease congestion of blood capillaries, while some pyknotic nuclei were still seen (Fig. 2D). Histopathological scores revealed significant histological alterations in the II /R group compared to sham and DIA groups (both *P* < 0.0001), while treatment with DIA showed significant improvement compared to II /R group (*P* < 0.0001) (Fig. 2E).

### Immunohistochemical results

#### Effect of DIA on caspase-1 immunoexpression

**Fig. 3:**
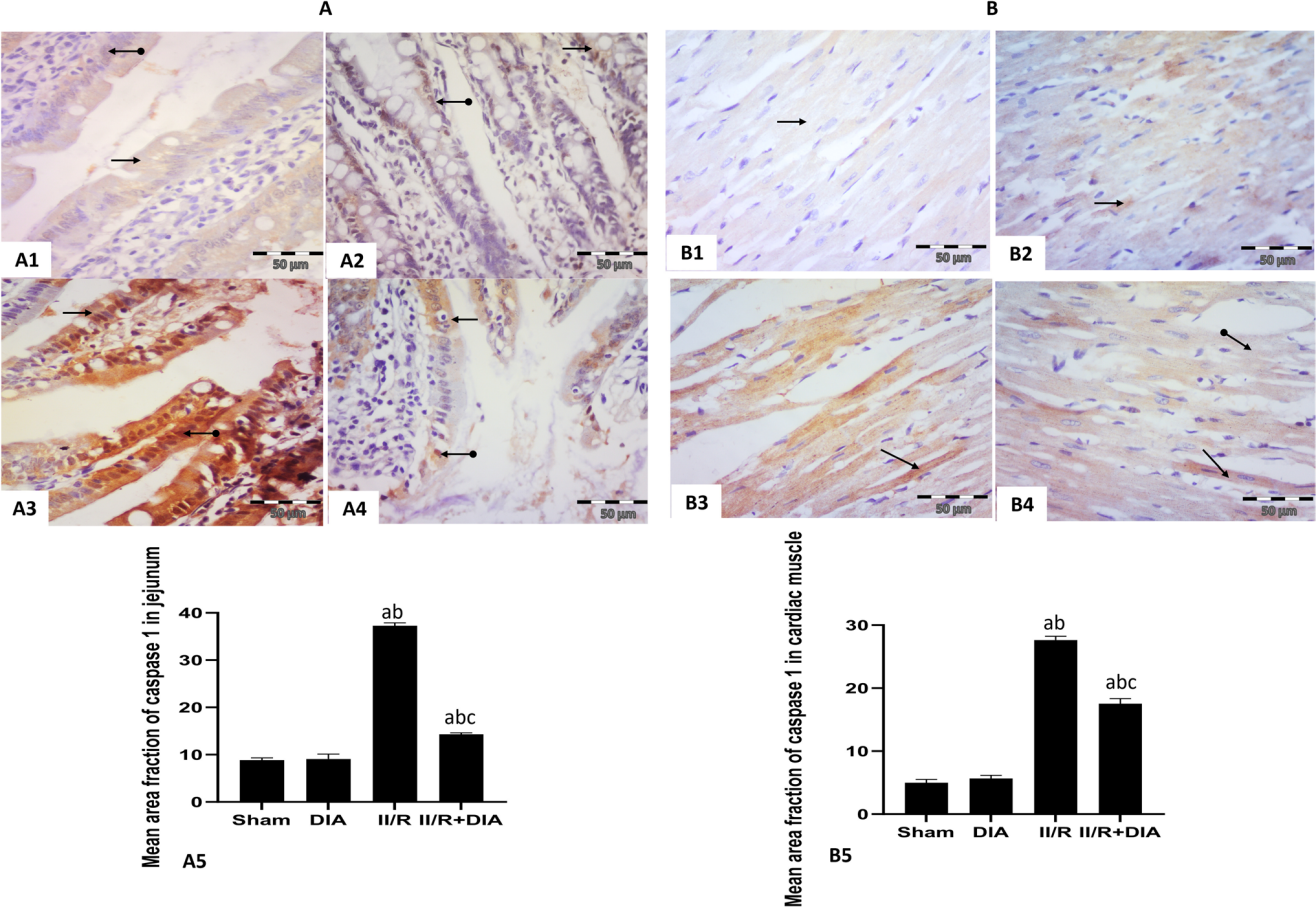
Representative photomicrographs of immunohistochemical analysis of caspase-1 protein expressions in paraffin-embedded rat jejunum (A1–4) and cardiac muscle tissues (B1–4): (**A1 **&** 2**) Sham and DIA groups respectively showed faint positive cytoplasmic (arrow) and nuclear (oval arrow) expression in epithelial cells lining the villi. (**A3**) II /R group showed intense positive cytoplasmic (arrow) and nuclear (oval arrow) expression in epithelial cells lining the villi. (**A4**) II / R+ DIA group showed moderate positive cytoplasmic (arrow) and nuclear (oval arrow) expression in epithelial cells lining the villi. (**B1 **&** 2**) Sham and DIA groups respectively showed faint positive sarcoplasmic (arrow) immune reaction in cardiac muscle fibers. (**B3**) II / R group showed intense positive sarcoplasmic (arrow) immune reaction in cardiac muscle fibers. (**B4**) II / R+ DIA group showed faint positive sarcoplasmic (oval arrow) immune reaction in most cardiac muscle fibers and moderate expression in some fibers (arrow). (anti-caspase-1 counter stained with hematoxylin X400). (**A5 **&** B5**). Values are representation of 10 observations (*n*=10) in each group as means ± S.E.M.  Results are considered significantly different when *P* < 0.05. ^a^Significant difference compared to sham group, ^b^Significant difference compared to DIA group, ^c^Significant difference compared to II/R group. DIA is diacerein, II/R is intestinal ischemia reperfusion induced group

Regarding jejunal sections, sham and DIA groups showed faint positive cytoplasmic and nuclear immune reaction in epithelial cells lining the villi (Fig. 3A1, 2). While the II /R group showed intense positive cytoplasmic and nuclear reaction (Fig. 3A3). On the other hand, II /R + DIA-treated group revealed moderate positive cytoplasmic and nuclear expression (Fig. 3A4).

Regarding cardiac muscle sections, sham and DIA groups showed faint positive sarcoplasmic immune reaction (Fig. 3B1, 2). On the contrary, II /R group showed intense positive sarcoplasmic immune reaction (Fig. 3B3), while co-administration of DIA had faint positive sarcoplasmic immune reaction in most cardiac muscle fibers and moderate expression in some fibers (Fig. 3B4).

The mean area fraction of caspase-1 immunoreactivity in jejunum and cardiac muscle showed that the II /R group had a significant increase compared to sham and DIA groups (all *P* < 0.0001), while DIA-treated group showed a significant decrease compared to the II /R group (both *P* < 0.0001) (Fig. 3A5, B5).

#### Effect of DIA on caspase-3 immunoexpression

**Fig. 4:**
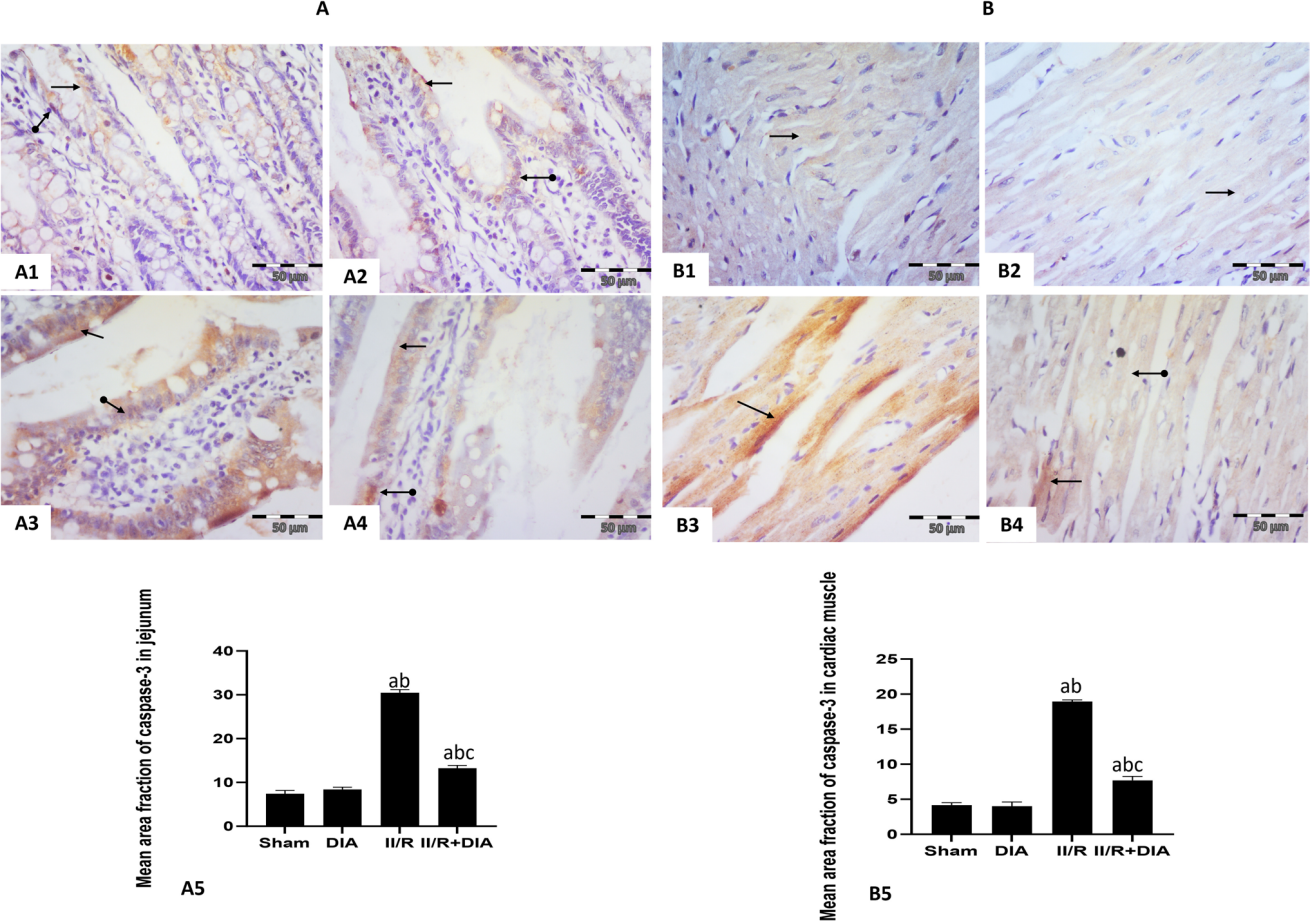
Representative photomicrographs of immunohistochemical analysis of caspase-3 protein expressions in paraffin-embedded rat jejunum (A1–4) and cardiac muscle tissues (B1–4): (**A1 **&** 2**) Sham and DIA groups respectively showing faint positive cytoplasmic (arrow) and nuclear (oval arrow) expression in epithelial cells lining the villi. (**A3**) II/R group showing intense positive cytoplasmic (arrow) and nuclear (oval arrow) expression in epithelial cells lining the villi. (**A4**) II /R+DIA group showing moderate positive cytoplasmic (arrow) and nuclear (oval arrow) expression in epithelial cells lining the villi. (**B1 **&** 2**) Sham and DIA groups respectively showing faint positive sarcoplasmic (arrow) immune reaction in cardiac muscle fibers. **(B3)** II / R group showing intense positive cytoplasmic (arrow) immune reaction in cardiac muscle fibers. (**B4**) II / R+DIA group showing faint positive cytoplasmic (oval arrow) immune reaction in most cardiac muscle fibers and moderate expression in few fibers (arrow). (anti-caspase-3 counter stained with hematoxylinX400). (**A5 **&** B5**). Values are representation of 10 observations (*n*=10) in each group as means ± S.E.M.  Results are considered significantly different when *P* < 0.05. ^a^Significant difference compared to sham group, ^b^Significant difference compared to DIA group, ^c^Significant difference compared to II/R group. DIA is diacerein, II/R is intestinal ischemia reperfusion induced group

Regarding jejunal sections, sham and DIA groups showed faint positive cytoplasmic and nuclear immune reaction in epithelial cells lining the villi (Fig. 4A1, 2), while the II /R group showed intense positive cytoplasmic and nuclear reaction (Fig. 4A3). However, II /R + DIA-treated group revealed moderate positive cytoplasmic and nuclear expression (Fig. 4A4).

Regarding cardiac muscle sections, sham and DIA groups respectively showed faint positive sarcoplasmic immune reaction (Fig. 4B1, 2). However, the II /R group exhibited intense positive sarcoplasmic immune reaction (Fig. 4B3). On the contrary, co-administration of DIA had faint positive sarcoplasmic immune reaction in most cardiac muscle fibers and moderate expression in few fibers (Fig. 4B4).

The mean area fraction of caspase-3 immunoreactivity in jejunum and cardiac muscle showed that the II /R group had a significant increase compared to sham and DIA groups (all *P* < 0.001), while DIA-treated group showed a significant decrease compared to the II /R group (both *P* < 0.0001) (Fig. 4A5, B5).

#### Effect of DIA on β-catenin immunoexpression

**Fig. 5:**
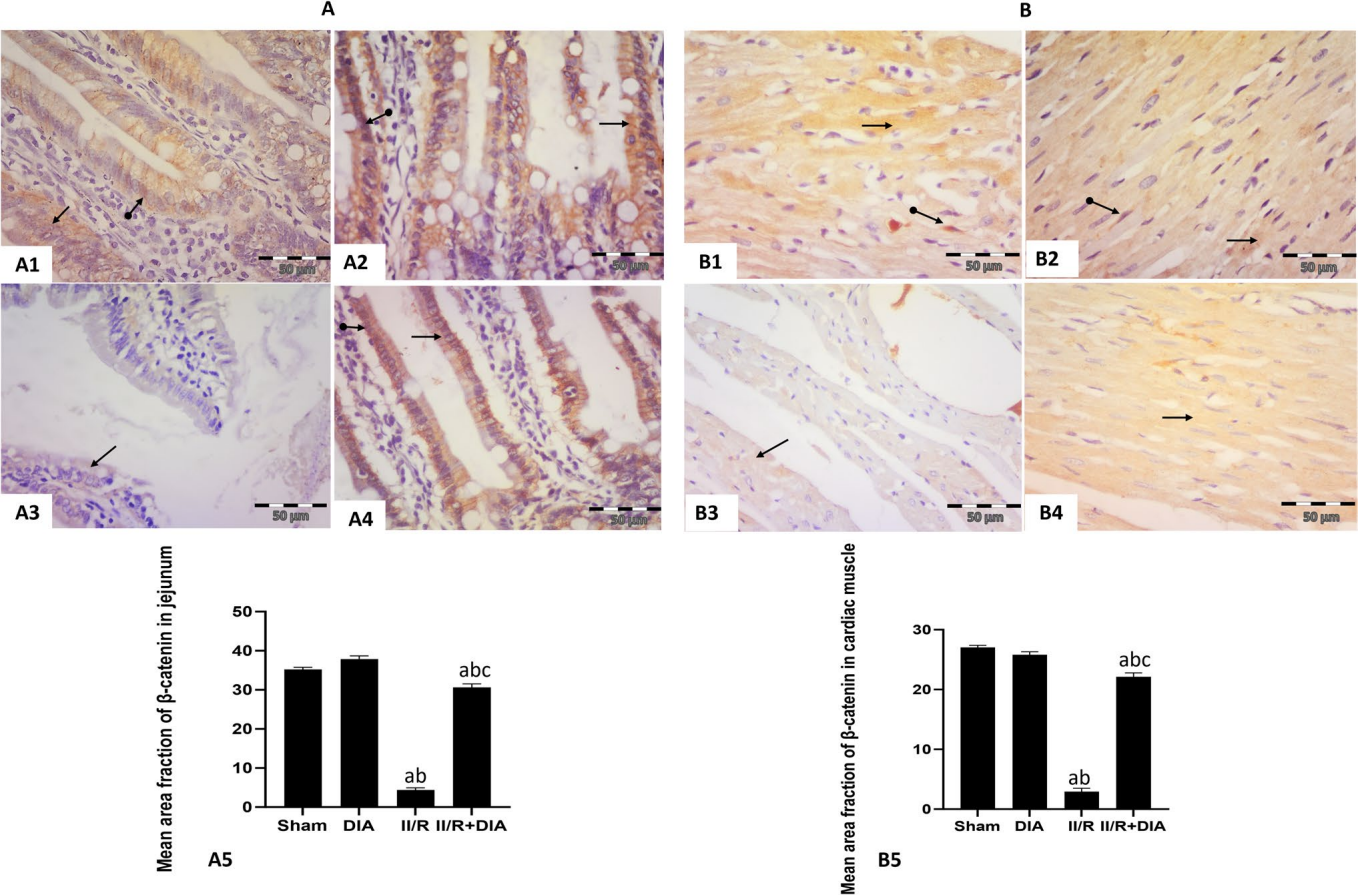
Representative photomicrographs of immunohistochemical analysis of β-catenin protein expressions in paraffin-embedded rat jejunum (A1–4) and cardiac muscle tissues (B1–4): (**A1 **&** 2**) Sham and DIA groups respectively showing intense positive cytoplasmic (arrow) and nuclear (oval arrow) expression in epithelial cells lining the villi. (**A3**) II / R group showing faint positive cytoplasmic (arrow) expression in epithelial cells lining the villi. (**A4**) II / R+DIA group showing moderate positive cytoplasmic (arrow) and nuclear (oval arrow) expression in epithelial cells lining the villi. (**B1 **&** 2**) Sham and DIA groups respectively showing positive sarcoplasmic (arrow) immune reaction in cardiac muscle fibers. (**B3**) II /R group showing faint positive sarcoplasmic (arrow) immune reaction in cardiac muscle fibers. (**B4**) II / R+DIA group showing positive cytoplasmic (arrow) immune reaction in cardiac muscle fibers. (anti- β-Catenin counter stained with hematoxylinX400). (**A5 **&** B5**). Values are representation of 10 observations (*n*=10) in each group as means ± S.E.M.  Results are considered significantly different when *P* < 0.05. ^a^Significant difference compared to sham group, ^b^Significant difference compared to DIA group, ^c^Significant difference compared to II/R group. DIA is diacerein, II/R is intestinal ischemia reperfusion induced group

Sham and DIA groups of jejunal sections showed intense positive cytoplasmic and nuclear reaction in epithelial cells lining the villi (Fig. 5A1, 2), while II /R group revealed faint positive cytoplasmic expression (Fig. 5A3). Co-treatment with DIA showed positive cytoplasmic and nuclear immune reaction (Fig. 5A3).

Sham and DIA groups of cardiac muscle sections showed intense positive sarcoplasmic immune reaction (Fig. 5B1, 2). However, II /R group revealed faint positive sarcoplasmic reaction (Fig. 5B3). On the other hand, DIA-treated group exhibited positive sarcoplasmic immune reaction (Fig. 5B4).

The mean area fraction of β-catenin immunoreactivity in jejunum and cardiac muscle showed that the II /R group had a significant decrease compared to sham and DIA groups (all *P* < 0.0001), while DIA-treated group showed a significant increase compared to the II /R group (both *P* < 0.0001) (Fig. 5A5, B5).

### Western blotting expression of IL-1β and Wnt1 (Fig. 6)

The expression of IL-1β as well as Wnt1 proteins in intestinal and cardiac tissues was assessed by Western blotting, and the findings revealed that IL-1β expression significantly increased but Wnt1 expression decreased in untreated II/R group compared with sham and DIA groups. However, co-administration of DIA significantly reduced IL-1β expression but raised Wnt1 expression if compared to untreated II/R group, *p* < 0.001.

**Fig. 6:**
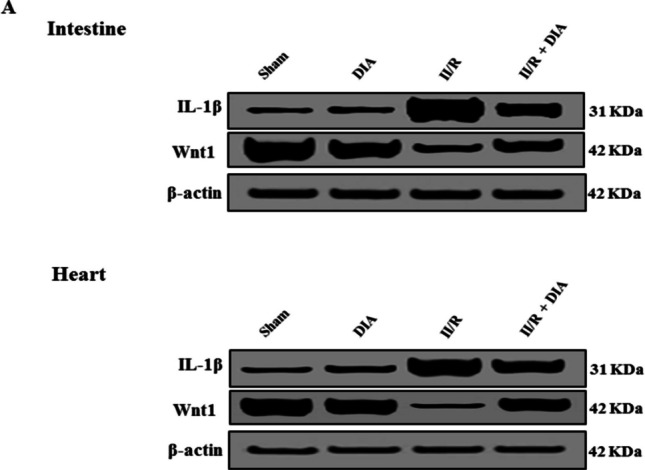

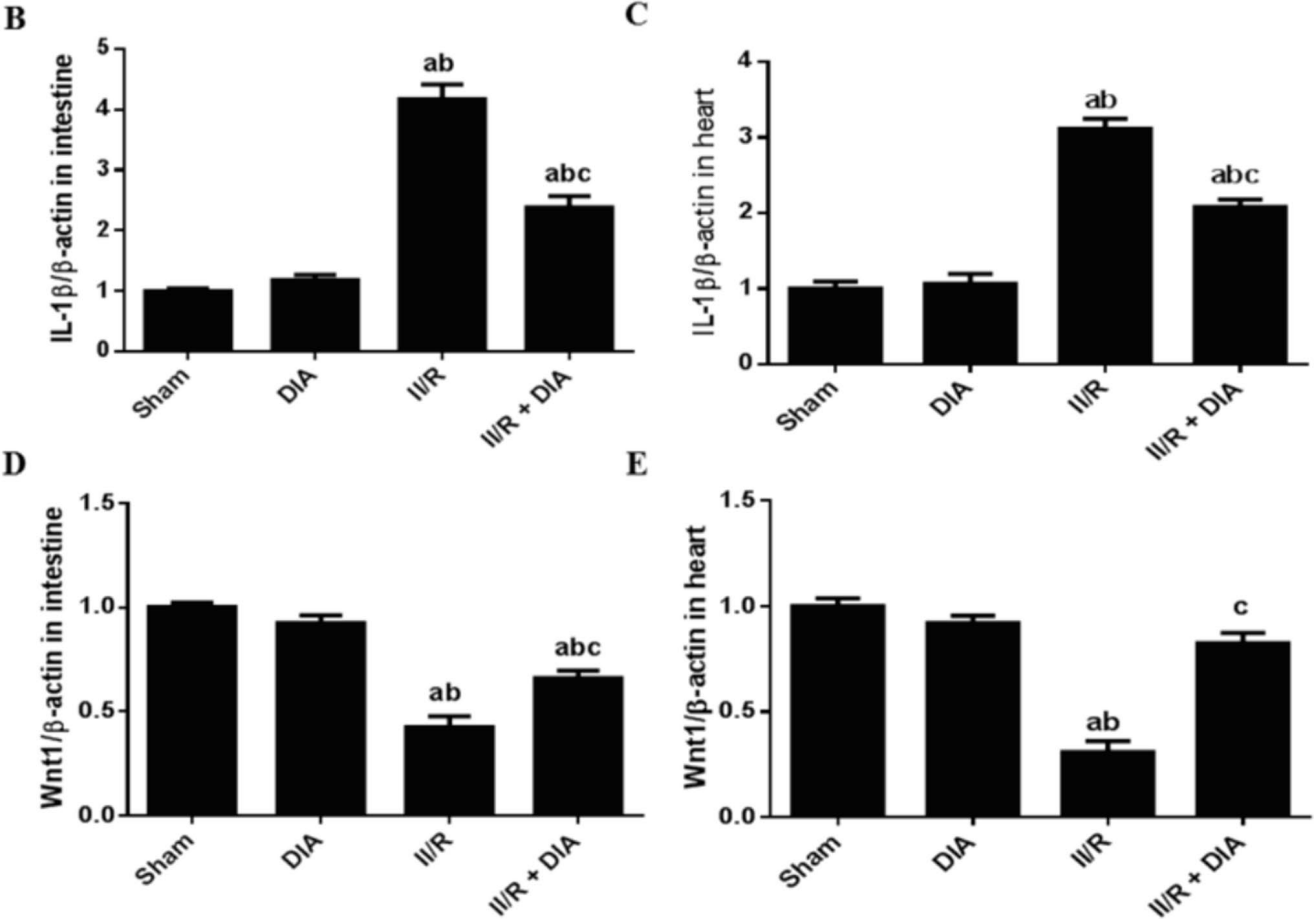
Western blot analysis showing the effect of DIA on the intestinal and cardiac expression of IL-1β and Wnt1 proteins. **A** Representative images of IL-1β, Wnt1, and β-actin proteins in different groups. **B**–**E** Following normalizing the bands in (**A**) to the appropriate internal reference β-actin, densitometric analysis was employed to assess protein expressions as fold change in respective to sham rats. Values are representation of mean ± S.E.M.  Results are considered significantly different when *P *< 0.05. ^a^Significant difference compared to sham group, ^b^Significant difference compared to DIA group. ^c^Significant difference compared to II/R group. DIA is diacerein, II/R is intestinal ischemia reperfusion induced group

## Discussion

Sudden ischemia is the most serious emergency situation and the intestine is considered one of the highly vulnerable organs to such damage that requires immediate surgical intervention or removal of the affected part but the accompanied harmful effect of ischemia is not controlled even after surgical manipulation (Chen et al. 2021; Archontakis‐Barakakis et al. 2025). Neonates and young children commonly suffer from intestinal ischemic injury during necrotizing enterocolitis, intestinal obstruction, and incarcerated hernia (Deng et al. 2022; Li et al. 2022). This guided us to search for an adequate adjuvant medical therapy besides the surgical intervention to control these hazards either in the intestine or in distant organs especially the heart tissue. In current model, we aimed to evaluate the possible role of DIA in II/R model in juvenile rats and study the different involved pathways including inflammasome/caspase-1/IL-1β and Wnt/β-catenin ones. Our findings showed significant increases in the levels of cardiac enzymes, MDA, IL-1β, caspase-3, and NF-κB in II/R group. On the opposite side, there are significant decreases in Wnt1, β-catenin, TAC, and GSH with marked histopathological changes in form of patchy erosion in the villi apical parts or totally sloughed in the lumen with disturbed cardiac muscle architecture and detached acidophilic areas were detected. However, DIA-treated group could regulate inflammasome/caspase-1/IL-1β and Wnt/β-catenin signaling cascades, diminish the elevated levels of cardiac enzymes and the inflammatory mediators with potent anti-oxidant and anti-apoptotic properties and obvious improvement in the histopathological features.

Until now, the mechanism of II/R-induced injury is not adequately understood but the excessive formation of ROS has a great role in mediating its damaging effect. These radicals attack all intracellular molecules and could stimulate further release of different inflammatory mediators including IL-1β, TNFα, and NF-κB leading to stimulation of apoptosis and initiating the squeals of cell death (Jia et al. 2020; Wang et al. 2021; Li et al. 2022). In addition, inadequate blood supply during intestinal ischemia diminishes tissue oxygenation and induces hypoxia with disturbance of the different inflammatory and apoptotic pathways (Grenz et al. 2012; Wang et al. 2021; Li et al. 2021, 2022; He et al. 2022; Ji et al. 2023; Zhang et al. 2024; Gültekin et al. 2024).

Oxidative stress is considered the corner stone in the pathogenesis of II/R injury. When oxygen supply is disrupted, xanthine dehydrogenase undergoes irreversible proteolytic alteration, by trypsin, and converts into xanthine oxidase, which has a high ability to form ROS. Evaluation of oxidative stress process and membrane lipid peroxidation is based mainly upon measuring MDA. On the opposite side, the primary defense mechanism against the released radicals is the intracellular antioxidants such as GSH which plays an essential role in mitigating the oxidative stress harmful effects. II/R leads to significant elevation of MDA levels, but diminishes tissue GSH and TAC serum levels because of the already excessively released free radicals (Paolillo et al. 2013; Korkmaz et al. 2020; Gokbulut et al. 2022). Besides that, apoptosis process serves a key role in mediating II/R induced injury and the damaged mitochondria could release the pro-apoptotic agents, triggering apoptosome formation and activates caspase-9 that is in turn enhances pro-caspase-3 and allows the formation of cleaved caspase-3 (Grenz et al. 2012; Li et al. 2022; Tan et al. 2023; Archontakis‐Barakakis et al. 2025; Cai et al. 2025). This is in accordance with our results that detected significant elevation of NF-κB; the triggering factor of inflammation and apoptosis with marked elevation of cleaved caspase-3 expression and it is the most important indicator of apoptosis.

Specific cardiac enzymes (CK-MB, LDH, Troponin I) are essential in evaluating the cardiac tissue function. During II/R, oxidative stress causes membrane lipid peroxidation and cell damage that is associated with the release of the intracellular cardiac enzymes followed by elevation of their levels (Paolillo et al. 2013; Korkmaz et al. 2020; Li et al. 2022; Gokbulut et al. 2022). In current research, there are significant elevations of CK-MB, LDH, and Tropinin I in untreated II/R group.

Wnt/β-catenin pathway also has the ability to interact with other key signaling pathways, creating an extensive network that collectively could regulate I/R injury including PI3K/Akt, TGF-β, NF-κB, or HIF-1α cascade, thereby regulating inflammation, apoptosis, and oxidative stress responses. Several researchers have found a decline in Wnt signaling activity after stroke onset. On the other side, the activators of Wnt/β-catenin pathway had encouraging therapeutic effects (Li et al. 2021; He et al. 2022; Zhang et al. 2024). This is in accordance with our data that revealed downregulation of Wnt/β-catenin cascade in intestinal ischemic group.

Moreover, increased vascular permeability of the vasculature during II/R causes fluid shift, contributing to hypovolemia and shock as nitric oxide imbalance and ROS production impair endothelial function, reduce coronary artery blood flow, and increase the risk of myocardial ischemia. Without proper diagnosis, appropriate surgical intervention, adequate management, and treatment, the risk of increased mortality and morbidity becomes exponentially high. Besides that, the adverse effects of intestinal ischemic injury are not restricted to the intestine itself and can cause several distinct organs injury especially the cardiac tissue (Doudakmanis et al. 2021; Ji et al. 2023; Gültekin et al. 2024).

Another essential contributing factors in mediating II/R is NLRP3 inflammasome and its downstream signaling cascade; inflammasome/caspase-1/IL-1β which have been proven to be vital molecules in ischemic conditions. IL-1β level highly increased after ischemia reperfusion injury and it activates macrophages in the early stages of intestinal injury as it has a potent modulating action on the intestinal epithelial tight junction causing increased intestinal permeability (Al-Sadi et al. 2012; Monaco et al. 2021; Xue et al. 2022; Ruera et al. 2022). In this way, blocking NLRP3 activation of related proteins is an effective approach against ischemic injury. DIA is an inhibitor of IL-1β which has been approved as an anti-inflammatory drug in treatment of osteoarthritis with potent inhibitory effect on inflammasome/caspase-1/IL-1β pathway (Shi et al. 2022; Refaie et al. 2022; Duan et al. 2024). Before reaching systemic circulation, DIA is converted in the liver into its active metabolite, rhein which has an ability to block IL-1β receptor and decreasing its number on cell membrane. Besides that, it can diminish IL-1β activity, synthesis and release. Thus, it prevents IL-1β-related downstream signaling pathways to reduce inflammatory gene transcription. This results in decreasing the production of inflammatory molecules and cytokines (Zhang et al. 2025).

Recently, DIA has gained a great potential insight as an anti-inflammatory and anti-oxidant agent (Zhang et al. 2025). Current data revealed that DIA treatment increased tissue levels of TAC and GSH associated with decreased MDA tissue levels, NF-κB, caspase-3, and cardiac enzymes with an obvious normalization of inflammasome/caspase-1/IL-1β and Wnt/β-catenin signaling cascades which is in agreement with others as DIA had an ability to control different forms of ischemia reperfusion-induced injury via regulating various inflammatory and apoptotic pathways such as cardiac, testicular, hepatic, brain, and renal ischemia reperfusion injury (Fouad et al. 2020; Tan et al. 2020; Agarwal et al. 2021; Silva et al. 2022; Wang et al. 2022; Abdelfattah et al. 2023; Samaha et al. 2023; El-Gohary et al. 2024; Abd Elrazik and Abd El Salam 2024). The tremendous effect of medical treatment in any model of ischemia reperfusion is during the acute attack of ischemia and before reperfusion as this is the most dangerous period trying to keep the tissue as possible. Thus, we focused mainly to evaluate this beneficial effect of DIA during acute intestinal ischemic injury simulating the clinical situation in patients. Further administration of the drug for long time after intestinal ischemia and following tissue damage will give less alleviating effect. Our current research highlights considering DIA in intestinal ischemia. Further studies should be carried out focusing on the possible essential ameliorative role of DIA in cases of intestinal ischemia and the associated remote organ damage especially cardiac tissue.

## Conclusion

DIA co-administration during II/R injury has the ability to ameliorate both the cardiac and intestinal tissue damages mostly via modulation of inflammasome/caspase-1/IL-1β and Wnt/β-catenin pathways with potent anti-oxidant, anti-inflammatory, and anti-apoptotic properties.

## Study limitations

The current model is limited by evaluating the effect of DIA on human cell culture and studying other molecular pathways. In addition, verifying the possible beneficial effect of DIA on patients of intestinal ischemic injury should be carried out to detect the suitable dose, regimen, and proper time for drug administration.

## Data Availability

Data is available upon request.

## Abbreviations

CK-MB: Creatine kinase-MB
DIA: Diacerein
GSH: Reduced glutathione
II/R: Intestinal ischemia reperfusion
IL-1β: Interleukin-1beta
LDH: Lactate dehydrogenase
MDA: Malondialdehyde
NF-κB: Nuclear factor kappa beta
ROS: Reactive oxygen species
TAC: Total antioxidant capacity
TNFα: Tumor necrosis factor alpha

## References

[CR1] Abd Elrazik NA, Abd El Salam ASG (2024) Diacerein ameliorates thioacetamide-induced hepatic encephalopathy in rats via modulation of TLR4/AQP4/MMP-9 axis. Metab Brain Dis 40:10. 10.1007/s11011-024-01457-x 39556255 10.1007/s11011-024-01457-x PMC11573817

[CR2] Abdelfattah AM, Mahmoud SS, EL-wafaey DI et al (2023) Diacerein ameliorates cholestasis-induced liver fibrosis in rat via modulating HMGB1/RAGE/NF-κB/JNK pathway and endoplasmic reticulum stress. Sci Rep 13:11455. 10.1038/s41598-023-38375-4 37454204 10.1038/s41598-023-38375-4 PMC10349817

[CR3] Abdel-Gaber SA, Mohammed RK, Refaie MMM (2018) Mechanism mediating the protective effect of diacerein in ischemia-reperfusion-induced testicular injury in rats. Life Sci 209:57–62. 10.1016/j.lfs.2018.07.060 30076921 10.1016/j.lfs.2018.07.060

[CR4] Agarwal V, Kaushik AS, Rehman M et al (2021) Interleukin-6 expression and its modulation by diacerein in a rat model of chronic stress induced cardiac dysfunction. Heliyon 7:e08522. 10.1016/j.heliyon.2021.e08522 34917808 10.1016/j.heliyon.2021.e08522 PMC8665349

[CR5] Akinrinde AS, Akinrinmade JF (2023) Multiple organ dysfunction syndrome: phytotherapeutic evidences from intestinal ischemia-reperfusion models. Curative and preventive properties of medicinal plants. Apple Academic Press, pp 141–163

[CR6] Ali GF, Hassanein EHM, Abdel-Reheim MA et al (2025) Involvement of IL‐6/JAK/STAT3/SOCS3, SIRT1, and cytoglobin signaling in diacerein protective effect against intestinal injury induced by methotrexate. J Biochem Mol Toxicol 39:e70339. 10.1002/jbt.70339 40488252 10.1002/jbt.70339

[CR7] Al-Sadi R, Guo S, Dokladny K et al (2012) Mechanism of interleukin-1β induced-increase in mouse intestinal permeability in vivo. J Interferon Cytokine Res 32:474–484. 10.1089/jir.2012.0031 22817402 10.1089/jir.2012.0031 PMC3464071

[CR8] Archontakis-Barakakis P, Mavridis T, Chlorogiannis D et al (2025) Intestinal oxygen utilisation and cellular adaptation during intestinal ischaemia–reperfusion injury. Clinical & Translational Med 15:e70136. 10.1002/ctm2.70136 10.1002/ctm2.70136 PMC11670310 39724463

[CR9] Bala M, Catena F, Kashuk J et al (2022) Acute mesenteric ischemia: updated guidelines of the World Society of Emergency Surgery. World J Emerg Surg 17:54. 10.1186/s13017-022-00443-x 36261857 10.1186/s13017-022-00443-x PMC9580452

[CR10] Bancroft JD, Layton C (2019) 10 - The hematoxylins and eosin. In: Suvarna SK, Layton C, Bancroft JD (eds) Bancroft’s Theory and Practice of Histological Techniques, 8th edn. Elsevier, pp 126–138. 10.1016/B978-0-7020-6864-5.00010-4

[CR11] Buege JA, Aust SD (1978) [30] Microsomal lipid peroxidation. Methods in enzymology. Elsevier, pp 302–310. 10.1016/S0076-6879(78)52032-6 10.1016/s0076-6879(78)52032-6 672633

[CR12] Cai Y, Wu Y, Guo Z et al (2025) Sevoflurane alleviates intestinal ischemia–reperfusion injury in aged mice. Med Gas Res 15:398–403. 10.4103/mgr.MEDGASRES-D-24-00033 39923136 10.4103/mgr.MEDGASRES-D-24-00033 PMC12054681

[CR13] Charan J, Biswas T (2013) How to calculate sample size for different study designs in medical research? Indian J Psychol Med 35:121–126. 10.4103/0253-7176.116232 24049221 10.4103/0253-7176.116232 PMC3775042

[CR14] Chen H, Guan B, Chen S et al (2021) Peroxynitrite activates NLRP3 inflammasome and contributes to hemorrhagic transformation and poor outcome in ischemic stroke with hyperglycemia. Free Radic Biol Med 165:171–183. 10.1016/j.freeradbiomed.2021.01.030 33515754 10.1016/j.freeradbiomed.2021.01.030

[CR15] Chen S, Xu H, Qin Y et al (2024) Nicotinamide adenine dinucleotide phosphate alleviates intestinal ischemia/reperfusion injury via Nrf2/HO-1 pathway. Int Immunopharmacol 143:113478. 10.1016/j.intimp.2024.113478 39471691 10.1016/j.intimp.2024.113478

[CR16] Constantin I, Tăbăran AF (2022) Dissection techniques and histological sampling of the heart in large animal models for cardiovascular diseases. JoVE 63809. 10.3791/63809 10.3791/63809 35786695

[CR17] Deng F, Lin Z-B, Sun Q-S et al (2022) The role of intestinal microbiota and its metabolites in intestinal and extraintestinal organ injury induced by intestinal ischemia reperfusion injury. Int J Biol Sci 18:3981–3992. 10.7150/ijbs.71491 35844797 10.7150/ijbs.71491 PMC9274501

[CR18] Doudakmanis C, Bouliaris K, Kolla C et al (2021) Bacterial translocation in patients undergoing major gastrointestinal surgery and its role in postoperative sepsis. WJGP 12:106–114. 10.4291/wjgp.v12.i6.106 34877025 10.4291/wjgp.v12.i6.106 PMC8611185

[CR19] Duan Y, Li Q, Wu J et al (2024) A detrimental role of endothelial S1PR2 in cardiac ischemia-reperfusion injury via modulating mitochondrial dysfunction, NLRP3 inflammasome activation, and pyroptosis. Redox Biol 75:103244. 10.1016/j.redox.2024.103244 38909407 10.1016/j.redox.2024.103244 PMC11254837

[CR20] El-aziz Fathy EA, Abdel-Gaber SA-W, Gaber Ibrahim MF et al (2024) Downregulation of IL-1β/p38 mitogen activated protein kinase pathway by diacerein protects against kidney ischemia/reperfusion injury in rats. Cytokine 176:156511. 10.1016/j.cyto.2024.156511 38290257 10.1016/j.cyto.2024.156511

[CR21] El-Gohary RM, Okasha AH, Abd El-Azeem AH et al (2024) Uncovering the cardioprotective potential of diacerein in doxorubicin cardiotoxicity: mitigating ferritinophagy-mediated ferroptosis via upregulating NRF2/SLC7A11/GPX4 axis. Antioxidants 13:493. 10.3390/antiox13040493 38671940 10.3390/antiox13040493 PMC11047461

[CR22] Fouad AA, Abdel-Aziz AM, Hamouda AAH (2020) Diacerein downregulates NLRP3/caspase-1/IL-1β and IL-6/STAT3 pathways of inflammation and apoptosis in a rat model of cadmium testicular toxicity. Biol Trace Elem Res 195:499–505. 10.1007/s12011-019-01865-6 31401744 10.1007/s12011-019-01865-6

[CR23] Gadde R, Xia J, Hameedi S et al (2025) Remote ischemic conditioning (RIC) decreases the incidence and severity of necrotizing enterocolitis (NEC) – validation in a large animal model. J Pediatr Surg 60:161957. 10.1016/j.jpedsurg.2024.161957 39368858 10.1016/j.jpedsurg.2024.161957

[CR24] Gokbulut P, Kuskonmaz SM, Koc G et al (2022) Evaluation of the effects of empagliflozin on acute lung injury in rat intestinal ischemia–reperfusion model. J Endocrinol Invest 46:1017–1026. 10.1007/s40618-022-01978-1 36495440 10.1007/s40618-022-01978-1

[CR25] Grenz A, Clambey E, Eltzschig HK (2012) Hypoxia signaling during intestinal ischemia and inflammation. Curr Opin Crit Care 18:178–185. 10.1097/MCC.0b013e3283514bd0 22322265 10.1097/MCC.0b013e3283514bd0 PMC3855266

[CR26] Gültekin Ç, Sayıner S, Çetinel Ş, Şehirli AÖ (2024) Outcomes of oxytocin treatment on intestinal ischemia-reperfusion injury in rats. Ankara Univ Vet Fak Derg 71:343–348. 10.33988/auvfd.1212713

[CR27] He J, Wo D, Ma E et al (2022) Huoxin pill prevents excessive inflammation and cardiac dysfunction following myocardial infarction by inhibiting adverse Wnt/β-catenin signaling activation. Phytomedicine 104:154293. 10.1016/j.phymed.2022.154293 35785558 10.1016/j.phymed.2022.154293

[CR28] Ji T, Chen M, Liu Y et al (2023) Artesunate alleviates intestinal ischemia/reperfusion induced acute lung injury via up-regulating AKT and HO-1 signal pathway in mice. Int Immunopharmacol 122:110571. 10.1016/j.intimp.2023.110571 37441813 10.1016/j.intimp.2023.110571

[CR29] Jia Y, Cui R, Wang C et al (2020) Metformin protects against intestinal ischemia-reperfusion injury and cell pyroptosis via TXNIP-NLRP3-GSDMD pathway. Redox Biol 32:101534. 10.1016/j.redox.2020.101534 32330868 10.1016/j.redox.2020.101534 PMC7178548

[CR30] Kielkopf CL, Bauer W, Urbatsch IL (2020) Bradford assay for determining protein concentration. Cold Spring Harb Protoc 2020:pdb.prot102269. 10.1101/pdb.prot102269 10.1101/pdb.prot102269 32238597

[CR31] Korkmaz A, Oyar EO, Yıldırım Z et al (2020) Application of vascular endothelial growth factor at different phases of intestinal ischemia/reperfusion: what are its effects on oxidative stress, inflammation and telomerase activity? Adv Clin Exp Med 29:1417–1424. 10.17219/acem/126297 33389832 10.17219/acem/126297

[CR32] Li S-S, Sun Q, Hua M-R et al (2021) Targeting the Wnt/β-catenin signaling pathway as a potential therapeutic strategy in renal tubulointerstitial fibrosis. Front Pharmacol 12:719880. 10.3389/fphar.2021.719880 34483931 10.3389/fphar.2021.719880 PMC8415231

[CR33] Li G, Wang S, Fan Z (2022) Oxidative stress in intestinal ischemia-reperfusion. Front Med 8:750731. 10.3389/fmed.2021.750731 10.3389/fmed.2021.750731 PMC8795364 35096858

[CR34] Monaco A, Ovryn B, Axis J, Amsler K (2021) The epithelial cell leak pathway. IJMS 22:7677. 10.3390/ijms22147677 34299297 10.3390/ijms22147677 PMC8305272

[CR35] Moron M, Depierre J, Mannervik B (1979) Levels of glutathione, glutathione reductase and glutathione S-transferase activities in rat lung and liver. Biochimica Et Biophysica Acta (BBA) - General Subjects 582:67–78. 10.1016/0304-4165(79)90289-7 760819 10.1016/0304-4165(79)90289-7

[CR36] Noordzij M, Dekker FW, Zoccali C, Jager KJ (2011) Sample size calculations. Nephron Clin Pract 118:c319–c323. 10.1159/000322830 21293154 10.1159/000322830

[CR37] Paolillo S, Rengo G, Pagano G et al (2013) Impact of diabetes on cardiac sympathetic innervation in patients with heart failure. Diabetes Care 36:2395–2401. 10.2337/dc12-2147 23530014 10.2337/dc12-2147 PMC3714495

[CR38] Refaie MMM, El-Hussieny M, Abdelraheem WM (2022) Diacerein ameliorates induced polycystic ovary in female rats via modulation of inflammasome/caspase1/IL1β and Bax/Bcl2 pathways. Naunyn-Schmiedebergs Arch Pharmacol 395:295–304. 10.1007/s00210-021-02175-2 34994825 10.1007/s00210-021-02175-2

[CR39] Refaie MMM, Amin EF, Hassan MN et al (2024) Sacubitril/valsartan protective effect on induced intestinal ischemia/reperfusion injury via immune modulation of IL6/STAT1 pathway. J Pharm Pharmacol 76:788–797. 10.1093/jpp/rgae031 38538077 10.1093/jpp/rgae031

[CR40] Refaie MMM, Mohammed HH, Abdel-Hakeem EA et al (2024) Cardioprotective role of diacerein in diabetic cardiomyopathy via modulation of inflammasome/caspase1/interleukin1β pathway in juvenile rats. Naunyn-Schmiedebergs Arch Pharmacol 397:5079–5091. 10.1007/s00210-023-02921-8 38224346 10.1007/s00210-023-02921-8 PMC11166746

[CR41] Ricardo-da-Silva FY, Fantozzi ET, Rodrigues-Garbin S et al (2021) Estradiol prevented intestinal ischemia and reperfusion-induced changes in intestinal permeability and motility in male rats. Clinics (Sao Paulo) 76:e2683. 10.6061/clinics/2021/e2683 33909827 10.6061/clinics/2021/e2683 PMC8050597

[CR42] Ruera C, Miculan E, Ducca G et al (2022) IL-1β blockade prevents cell death and mucosal damage of the small intestine in a model of sterile inflammation. Immunol Lett 251:56–62. 10.1016/j.imlet.2022.10.006 36309159 10.1016/j.imlet.2022.10.006

[CR43] Samaha MM, Nour OA, Sewilam HM, El-Kashef DH (2023) Diacerein mitigates adenine-induced chronic kidney disease in rats: focus on TLR4/MYD88/TRAF6/NF-κB pathway. Life Sci 331:122080. 10.1016/j.lfs.2023.122080 37690574 10.1016/j.lfs.2023.122080

[CR44] Schneider CA, Rasband WS, Eliceiri KW (2012) NIH image to ImageJ: 25 years of image analysis. Nat Methods 9:671–675. 10.1038/nmeth.2089 22930834 10.1038/nmeth.2089 PMC5554542

[CR45] Shi M, Chen J, Liu T et al (2022) Protective effects of remimazolam on cerebral ischemia/reperfusion injury in rats by inhibiting of NLRP3 inflammasome-dependent pyroptosis. DDDT 16:413–423. 10.2147/DDDT.S344240 35210755 10.2147/DDDT.S344240 PMC8863189

[CR46] Silva RCL, Sasso-Cerri E, Cerri PS (2022) Diacerein-induced interleukin-1β deficiency reduces the inflammatory infiltrate and immunoexpression of matrix metalloproteinase-8 in periodontitis in rat molars. J Periodontol 93:1540–1552. 10.1002/JPER.21-0375 35184279 10.1002/JPER.21-0375

[CR47] Suvarna KS, Layton C, Bancroft JD (2018) Bancroft’s theory and practice of histological techniques E-Book. Elsevier health sciences; p, London, p 654

[CR48] Tan Y, Zhang Z, Zheng C et al (2020) Mechanisms of diabetic cardiomyopathy and potential therapeutic strategies: preclinical and clinical evidence. Nat Rev Cardiol 17:585–607. 10.1038/s41569-020-0339-2 32080423 10.1038/s41569-020-0339-2 PMC7849055

[CR49] Tan C, Norden PR, Yu W et al (2023) Endothelial FOXC1 and FOXC2 promote intestinal regeneration after ischemia-reperfusion injury. EMBO Rep 24:e56030. 10.15252/embr.202256030 37154714 10.15252/embr.202256030 PMC10328078

[CR50] Torina AG, Reichert K, Lima F et al (2015) Diacerein improves left ventricular remodeling and cardiac function by reducing the inflammatory response after myocardial infarction. PLoS One 10:e0121842. 10.1371/journal.pone.0121842 25816098 10.1371/journal.pone.0121842 PMC4376692

[CR51] Wang Y, Wen J, Almoiliqy M et al (2021) Sesamin protects against and ameliorates rat intestinal ischemia/reperfusion injury with involvement of activating Nrf2/HO-1/NQO1 signaling pathway. Oxid Med Cell Longev 2021:5147069. 10.1155/2021/5147069 34630849 10.1155/2021/5147069 PMC8494576

[CR52] Wang M, Luo W, Yu T et al (2022) Diacerein alleviates Ang II-induced cardiac inflammation and remodeling by inhibiting the MAPKs/c-Myc pathway. Phytomedicine 106:154387. 10.1016/j.phymed.2022.154387 36027716 10.1016/j.phymed.2022.154387

[CR53] Xue S, Xue Y, Dou D et al (2022) Kui jie tong ameliorates ulcerative colitis by regulating gut microbiota and NLRP3/caspase-1 classical pyroptosis signaling pathway. Dis Markers 2022:1–15. 10.1155/2022/2782112 10.1155/2022/2782112 PMC9273439 35832643

[CR54] Zhang M, Liu Q, Meng H et al (2024) Ischemia-reperfusion injury: molecular mechanisms and therapeutic targets. Sig Transduct Target Ther 9:12. 10.1038/s41392-023-01688-x 10.1038/s41392-023-01688-x PMC10772178 38185705

[CR55] Zhang B, Cao T, Cao J et al (2025) Injectable oxidized hyaluronic acid/quaternized chitosan hydrogel encapsulated with diacerein microsphere for improved osteoarthritis treatment. Int J Biol Macromol 319:145395. 10.1016/j.ijbiomac.2025.145395 40541890 10.1016/j.ijbiomac.2025.145395

